# Correction to “Combination of an Engineered *Lactococcus lactis* Expressing CXCL12 With Light‐Emitting Diode Yellow Light as a Treatment for Scalded Skin in Mice”

**DOI:** 10.1111/1751-7915.70296

**Published:** 2025-12-26

Zhao, X., S. Li, J. Ding, J. Wei, P. Tian, H. Wei, and T. Chen. 2021. “Combination of an Engineered *Lactococcus lactis* Expressing CXCL12 With Light‐Emitting Diode Yellow Light as a Treatment for Scalded Skin in Mice.” *Microbial Biotechnology* 14, no. 5: 2090–2100.

In Figure 2A, two unintentional image issues were identified: one is an unintentional reuse of images from the same HE section; the other is an inadvertent swapping of image positions representing two different time points within the same experimental group. These have been corrected in the version of Figure 2 shown below. It is important to note that these corrections do not affect the core conclusions of this study in any way.

**FIGURE 2:**
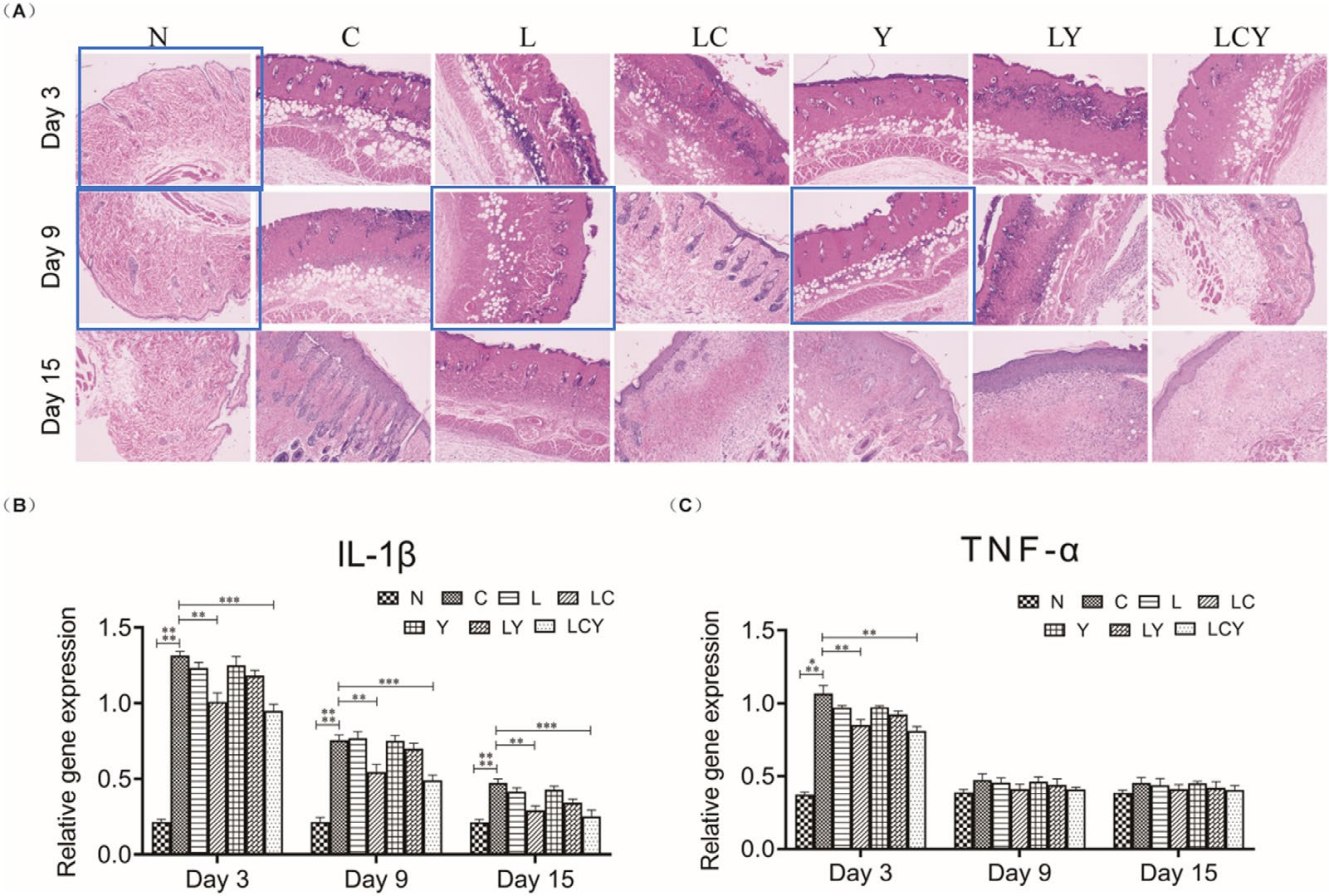
Effect of engineered combined with light on inflammation in the wound‐healing process of scalded mice. (A) Stained with H&E stain of the wounds in the C, L, LC, Y, LY and LCY groups at 3, 9 and 15 days post‐scald (*n* = 4 per group). Magnification: ×100. (B) The gene levels of IL‐1β and (C) TNF‐α in skin tissues of experimentally scalded mice (*n* = 4 per group; one‐way ANOVA Kruskal–Wallis test, *p < 0.05, **p < 0.001). The N group was not scalded, C group treated with saline, L group treated with MG1363, LC group treated with MG1363‐pMG36e‐mCXCL12, Y group treated with LED yellow light irradiation, LY group treated with MG1363+LED yellow light irradiation and LCY group treated with MG1363‐pMG36e‐mCXCL12+LED yellow light irradiation.

We apologize for this error.

